# Antitumor Effect of Zhihuang Fuzheng Soft Capsules on Tumor-Bearing Mice

**DOI:** 10.1155/2016/7503726

**Published:** 2016-07-04

**Authors:** Yanyan Bao, Xin Pan, Yahong Jin, Yingjie Gao, Xiaolan Cui

**Affiliations:** Biosafety Laboratory, Institute of Chinese Materia Medica, China Academy of Chinese Medical Sciences, Beijing 100700, China

## Abstract

Chinese medicines (CMs) have been shown to have some advantages in preventing and controlling tumors. In this study, we investigated the antitumor effect of ZFSC by establishing a mouse model of HT-1080, A-549, and HCT-8 tumors. The result showed that tumor volumes of HT-1080 tumor-bearing nude mice in ZFSC low, medium, and high dose groups were lower significantly compared to the model group, and the high dose ZFSC showed the best antitumor effect. Tumor volumes of A-549 tumor-bearing nude mice in ZFSC low, medium, and high dose groups were lower significantly compared to the model group and showed a good dose-response relationship. There was no significant effect on human colon cancer, although inhibition trends disappeared in the bar chart. In order to verify the immunomodulatory effect of ZFSC, ELISA was used to analyze serums IL-2, TNF-*α*, and IFN in spleens. The results showed that ZFSC could enhance the immune function of tumor-bearing mice. ZFSC reduced IFN-*γ* and TNF-*α* content in the serum of HT-1080 tumor-bearing mice and inhibit PD1 and PDL1 and suggested that the antitumor mechanism of ZFSC on human fibrosarcoma could be attributed to inhibition of the PDL1/PD1 pathway.

## 1. Introduction

Some traditional Chinese prescriptions have been identified as effective antitumor drugs in patients with cancer. Among the existing antitumor drugs, Chinese medicines (CMs) have many advantages, such as multitarget, multichannel, wide antitumor spectrum, low toxicity and side effects, long survival time, pain reduction, and high quality of life.

CMs have been shown to have some advantages in preventing and controlling tumors [1]. For example, artemisinin can selectively induce apoptosis in pancreatic tumor cells [2]. Furthermore, Fuzheng Yiliu Decoction inhibited HepG2 cells* in vitro* [3]. Finally, Jianpi Jiedu Recipe was found to affect reversion of P-glycoprotein-mediated multidrug resistance through the COX-2 pathway in colorectal cancer [4].

In tumors, the expression of programmed death ligand 1 (PDL1) on the cell surface interacts with its receptor, programmed death 1 (PD1) on T cells, which leads to the apoptosis of tumor antigen-specific T cells. This is the main mechanism of PDL1/PD1 signal-mediated tumor immune escape [5]. PDL1 is expressed in many tumor-associated antigen presenting cells (APCs) and tumor cells and can inhibit T cell proliferation. Negative regulation of PDL1/PD1 could reduce release of T cells inflammatory factors such as IL-2, IFN-*γ*, and TNF-*α* [6]. Blocking PDL1/PD1 signaling could be used in immunotherapy of human tumors. Iwai et al. found that an anti-PDL1 mAb could inhibit local tumor growth in PDL1-P815 tumor-bearing mice with an effective remission rate [7]. Hence, PDL1/PD1 signaling pathway is expected to become a new strategy for tumor immunotherapy.

Zhihuang Fuzheng Soft Capsule (ZFSC) is composed of* Ganoderma *spore oil,* Ganoderma *extract*, Phellinus igniarius *extract, and* Panax notoginseng saponins*.* Ganoderma* and* Phellinus igniarius *are CMs that may enhance the immune system to help fight cancer [8, 9].* Panax notoginseng saponin* is CM that may be called Huoxue Quyu. In this study, we investigated the antitumor effects of ZFSC.

## 2. Methods

### 2.1. Component and Production Process of Zhihuang Fuzheng Soft Capsules

Zhihuang Fuzheng Soft Capsules (ZFSC) were provided by the Third Hospital of Beijing Armed Police Corps (Beijing, China). ZFSC is composed of* Ganoderma *spore oil,* Ganoderma *extract,* Phellinus igniarius *extract, and* Panax notoginseng saponins*. According to the clinical dosage of 3.0 g/60 kg/day, the equivalent dosage for mice was set at 550 mg/kg/day. In this study, we used 275, 550, and 1100 mg/kg/day.

### 2.2. Positive Drug

Zhenqi Fuzheng Particles (positive control drugs) were produced by Gansu Fuzheng Pharmaceutical Technology Co., Ltd. Zhenqi Fuzheng Particles may enhance the immune system to help fight cancer. According to the clinical dosage of 30 g/60 kg/day, the equivalent dosage of mice was set at 5.5 g/kg/day.

### 2.3. Experimental Animals

Male and female BALB/c-nude mice (SPF-class, 18–20 g) were provided by Beijing Vital River Laboratory Animal Technology Co., Ltd. All animal experiments were performed according to the Guide for the Care and Use of Laboratory Animals of China Academy of Traditional Chinese Medicine.

### 2.4. Cell Lines Culture and Reagents

Human fibrosarcoma HT-1080 cells, human lung adenocarcinoma A-549 cells, and human colon cancer HCT-8 cells were purchased from the Peking Union Cell Line Resource Center. HT-1080 cells were cultured in Minimum Essential Medium (MEM, HyClone, USA) containing 10% (v/v) fetal bovine serum (FBS, Gibco, USA). A-549 and HCT-8 cells were cultured in Dulbecco's Modified Eagle Medium (DMEM, HyClone, USA) containing 10% (v/v) fetal bovine serum (FBS, Gibco, USA). All cells were cultured in a humidified atmosphere of 95% air and 5% CO_2_ at 37°C.

### 2.5. Model of Tumor-Bearing Mice

Cell lines were digested with Trypsin-EDTA (0.25%, Gibco, USA), the supernatant was discarded after centrifugation (1000 rpm), and the cells were resuspended in culture medium. Cells were diluted to approximately 1.5 × 10^7^ cells/mL, and 0.4 mL HT-1080, A-549, or HCT-8 cell suspension was injected into the right armpit of each nude mouse (three mice for each tumor). When the average size of the tumors was 1000 mm^3^, they were homogenized in physiological saline (0.25 g/mL) and reinjected into three more mice. Tumors were passaged at least three times* in vivo*.

The fourth generation of tumors was injected into 40 nude mice, and mice were randomly divided into five groups: model control group, positive drug control group, 275 mg/kg ZFSC group, 550 mg/kg ZFSC group, and 1100 mg/kg ZFSC group. Mice were given treatments orally for 2 weeks starting 1 day after inoculation. The model control group was given distilled water.

### 2.6. Measurement of Tumor Volume

Tumor growth was observed daily and tumor size was measured once every other day with calipers as soon as the visible tumor appeared. Tumor volume was calculated with the following formula:

(1) $$\begin{array}{c} \text{Tumor volume (TV)}=\frac{1}{2}\times {ab}^{2}. \end{array}$$

In this formula, “*a*” represents the longest diameter of tumor and “*b*” represents the shortest diameter of tumor.

### 2.7. Measurement of Tumor Inhibition Rate

After the last administration, blood was collected from the eye vein and centrifuged to obtain serums. Tumors were weighted and spleens were removed and stored at −80°C until use. The tumor inhibition rate was calculated with the following formula:

(2) Tumor inhibition rate (%)=tumor weight (model)−tumor weight (drug)tumor weight (model)×100%.

### 2.8. ELISA

IL-2, IFN-*γ*, and TNF-*α* content in serum were analyzed with ELISA kits according to the manufacturer's instructions. Spleens homogenates were prepared with phosphate buffer solution (PBS, HyClone, USA), and CD4/CD8 value, NK cell activity, and PD1 and PDL1 content in spleens were analyzed with ELISA according to the manufacturer's instructions. The double-antibody sandwich method was used to analyze the NK cell activity. Briefly, a microplate was coated with a mouse NK antibody, and then spleen cell homogenates and HRP-labeled NK cell antibodies were successively added to the microplate. Finally, the substrate 3,3′,5,5′-Tetramethylbenzidine (TMB) was added to the microplate and a change in color was observed. Absorbance (OD value) was determined at 450 nm. The depth and yellow color were positively correlated with NK cell activity.

### 2.9. Statistical Analysis

All data are expressed as the mean ± standard deviation (SD). ANOVA was used for the determination of statistical significance (*p* < 0.05). For the pairwise comparison of groups, the LSD test (homogeneity of variance) or Dunnett's *t*3 test (heterogeneity of variance) was applied. 

## 3. Results

### 3.1. Inhibitory Effect of ZFSC on Human Fibrosarcoma, Human Lung Adenocarcinoma, and Human Colon Cancer

**Figure 1:**
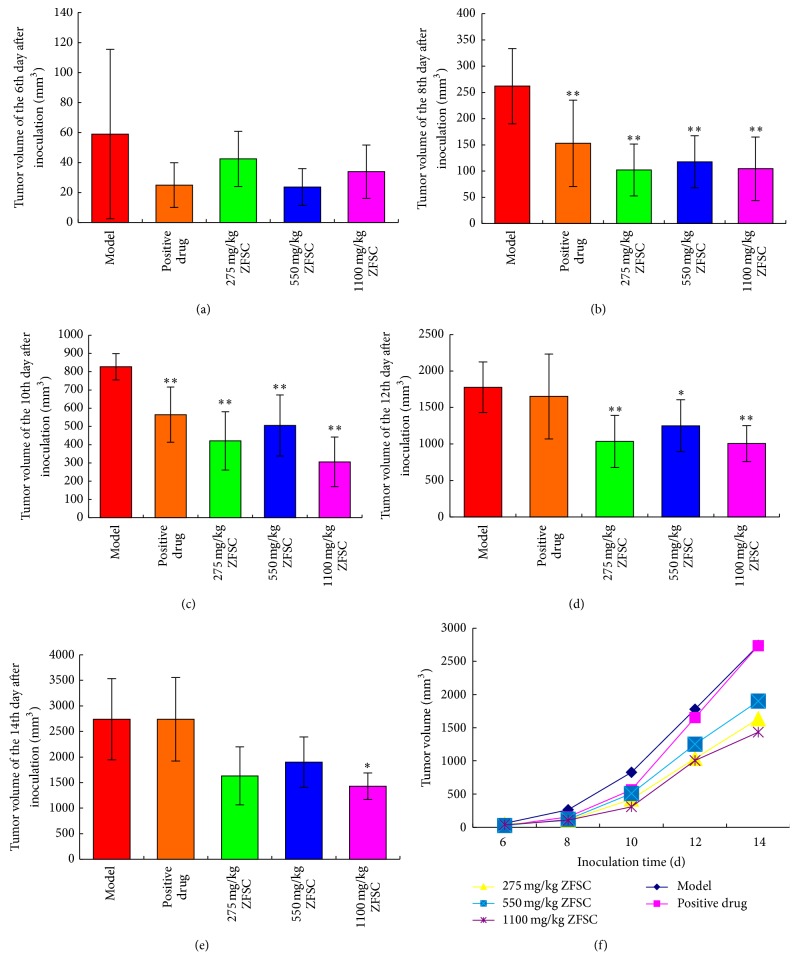
Tumors value of HT-1080 tumor-bearing mice. Notes: (a)–(e), respectively, present the tumors value of the 6th, 8th, 10th, 12th, and 14th day after inoculation, 275 mg/kg ZFSC, 550 mg/kg ZFSC, and 1100 mg/kg ZFSC could significantly inhibit human fibrosarcoma from the 8th day to the 12th day after inoculation, but only 1100 mg/kg ZFSC could significantly inhibit human fibrosarcoma at the 14th day after inoculation, and the result revealed that 1100 mg/kg ZFSC has the best inhibitory effect on human fibrosarcoma although 275 mg/kg ZFSC and 550 mg/kg ZFSC also play a role. (f) presents tumors value-time curve of each group. ^*∗*^ *p* < 0.05, ^*∗∗*^ *p* < 0.01, and *n* = 8.

**Figure 2:**
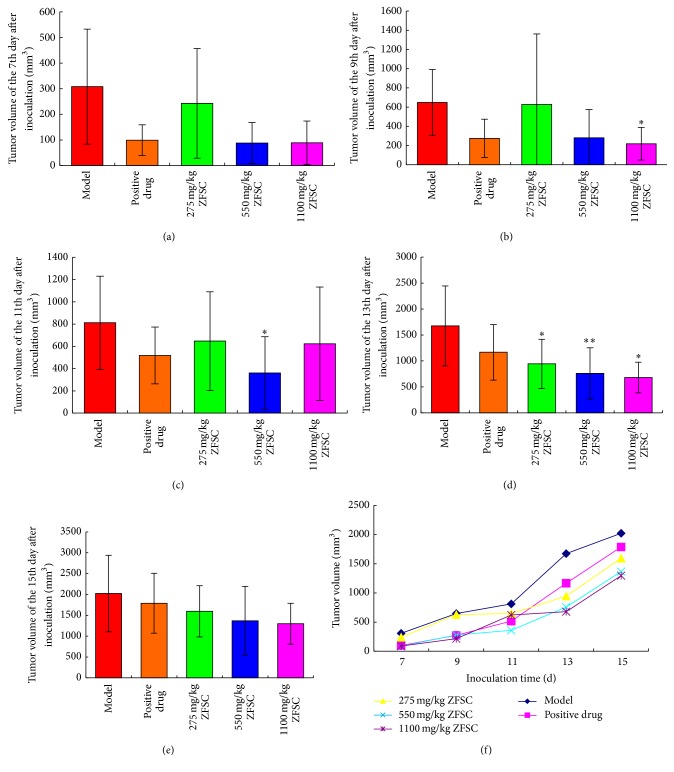
Tumors value of A-549 tumor-bearing mice. Notes: (a)–(e), respectively, present the tumors value of the 7th, 9th, 11th, 13th, and 15th day after inoculation, the significant inhibitory effect of 1100 mg/kg ZFSC on human lung adenocarcinoma appeared momently at the 9th day and the 13th day after inoculation, from the 11th day to the 13th day after inoculation 550 mg/kg ZFSC showed significant inhibitory effect on human lung adenocarcinoma, and at the 13th day after inoculation 275 mg/kg ZFSC also revealed significant inhibitory effect on human lung adenocarcinoma. The results showed that 1100 mg/kg ZFSC has better inhibitory effect on human fibrosarcoma. (f) presents tumors value-time curve of each group. ^*∗*^ *p* < 0.05, ^*∗∗*^ *p* < 0.01, and *n* = 8.

**Figure 3:**
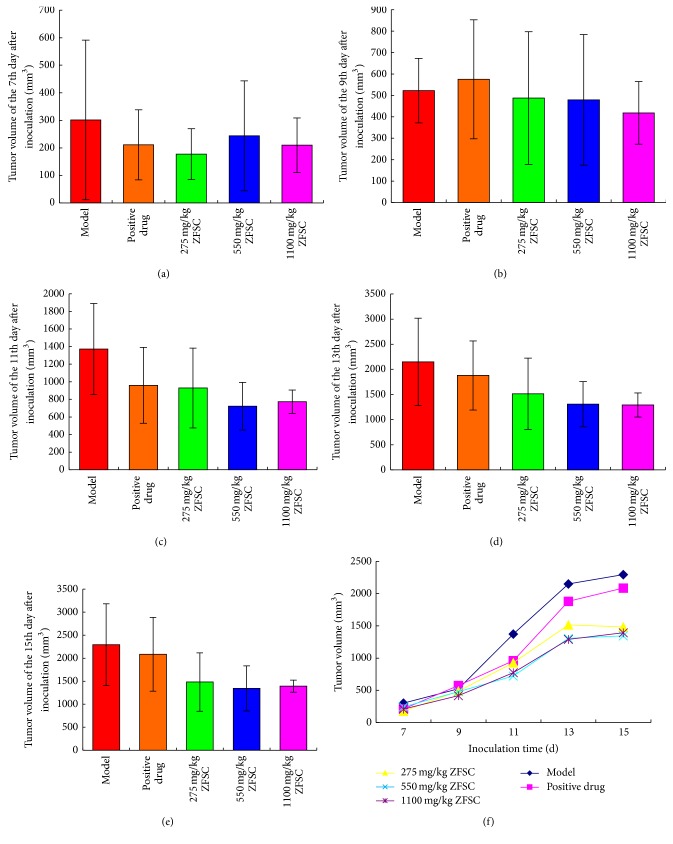
Tumors value of HCT-8 tumor-bearing mice. Notes: (a)–(e), respectively, present the tumors value of the 7th, 9th, 11th, 13th, and 15th day after inoculation; there were no significant effects of 275 mg/kg ZFSC, 550 mg/kg ZFSC, and 1100 mg/kg ZFSC on human colon cancer, although inhibition trends disappeared in the bar chart. (f) presents tumors value-time curve of each group. *n* = 8.

ZFSC could significantly inhibit the growth of human fibrosarcoma and human lung adenocarcinoma cells (*p* < 0.05), but not human colon cancer cells (*p* > 0.05). Dosages of 275 mg/kg ZFSC, 550 mg/kg ZFSC, and 1100 mg/kg ZFSC could significantly inhibit human fibrosarcoma from the 8th to the 12th day, while the inhibition of 1100 mg/kg ZFSC could continue to the 14th day. The tumor inhibition rates of the three dosages were 36.3%, 20.4%, and 40.2%, respectively (Table 1, Figure 1). The inhibitory effects of 1100 mg/kg ZFSC on human lung adenocarcinoma were observed on the 9th and 13th day, the inhibitory effects of 550 mg/kg ZFSC on human lung adenocarcinoma were observed on the 11th and 13th day, and the inhibitory effects of 275 mg/kg ZFSC on human lung adenocarcinoma were observed on the 13th day. The tumor inhibition rates of the three dosages were 15.0%, 24.0%, and 28.4% (Table 2, Figure 2). There were no significant effects of 275 mg/kg ZFSC, 550 mg/kg ZFSC, or 1100 mg/kg ZFSC on human colon cancer within 15 days. The tumor inhibition rates of the three dosages were 11.8%, 17.6%, and 15.6%, respectively (Table 3, Figure 3).

Positive drug (Zhenqi Fuzheng Particles) may enhance the immune system to help fight cancer. In this study, compared with the positive control group, the inhibitory effects of ZFSC on human fibrosarcoma, human lung adenocarcinoma, and human colon cancer were better. The tumor inhibition rates of ZFSC were better compared to positive drug on the three tumors.

These results indicate that the inhibition rate of the 1100 mg/kg ZFSC dosage was the best and the antitumor effect of ZFSC on human fibrosarcoma was better than that on human lung adenocarcinoma.

### 3.2. Effect of ZFSC on IL-2, TNF-*α*, and IFN-*γ* Serum Content

Serum was obtained and analyzed with IL-2, TNF-*α*, and IFN-*γ* by ELISA. IL-2 content was not altered by ZFSC in HT-1080 tumor-bearing mice (*p* > 0.05), but IFN-*γ* content was significantly lower in the 1100 mg/kg and 550 mg/kg ZFSC groups compared with the control group (*p* < 0.05). TNF-*α* content was significantly lower in the 1100 mg/kg ZFSC groups compared with the control group (*p* < 0.05) (Table 4). The serum of A-549 tumor-bearing mice had significantly higher IL-2 content in each ZFSC group and IFN-*γ* content was significantly lower in the 275 mg/kg and 550 mg/kg ZFSC groups compared with the control group (*p* < 0.05). The TNF-*α* content was not significantly altered by ZFSC (*p* > 0.05) (Table 5).

### 3.3. Effect of ZFSC on CD4/CD8 Value, NK Cell Activity, and PD1 and PDL1 Spleen Content

Spleens were homogenized and CD4/CD8 value, NK cell activity, PD1 and PDL1 contents were analyzed with ELISA. The CD4/CD8 value and NK cell activity were not affected by ZFSC in HT-1080 tumor-bearing mice (*p* > 0.05), but PD1 and PDL1 content were significantly lower compared with the control group (*p* < 0.05) (Table 6). Therefore, the anti-HT-1080 tumor mechanism of ZFSC could be attributed to the inhibition of the PDL1/PD1 pathway. The CD4/CD8 value, NK cell activity, and PD1 and PDL1 contents in spleens of A-549 tumor-bearing mice were not changed by ZFSC administration (Table 7).

## 4. Discussion

Surgery, radiation therapy, and chemotherapy are the methods commonly used to treat cancer in Western medicine. Although these therapies are constantly improving, they often include side effects, such as weight loss, nausea, vomiting, hair loss, and immune dysfunction. Therefore, comprehensive treatment plays an important role in tumor treatment, and CM is a significant part of comprehensive tumor treatment. CM can decrease the toxicity of chemotherapy, radiotherapy, and target therapy, enhance the antitumor effects of these therapies, alleviate clinical symptoms, stabilize tumor size, strengthen the body constitution, increase survival, relieve complications, and regulate the immune system [10].

ZFSC is composed of* Ganoderma *spore oil,* Ganoderma *extract*, Phellinus igniarius *extract, and* Panax notoginseng saponins *and is used as an adjuvant drug in tumor therapy. ZFSC is able to inhibit tumor growth and enhance immune function in tumor patients. In this study, we investigated the antitumor effect of ZFSC by establishing a mouse model of HT-1080, A-549, and HCT-8 tumors. Tumor growth was observed daily and tumor size was measured every other day as soon as the visible tumor appeared. Throughout the observation period, tumor volumes of HT-1080 tumor-bearing nude mice in ZFSC low, medium, and high dose groups were lower significantly compared to the model group, and the high dose ZFSC showed the best antitumor effect. Meanwhile, tumor inhibition rates of ZFSC low, medium, and high dose groups were 36.3%, 20.4%, and 40.2%, respectively, which also verified the above result. Tumor volumes of A-549 tumor-bearing nude mice in ZFSC low, medium, and high dose groups were lower significantly compared to the model group and showed a good dose-response relationship. Tumor inhibition rates of ZFSC low, medium, and high dose groups were 15.0%, 24.0%, and 28.4%, respectively, which also confirmed this point. There was no significant effect on human colon cancer, although inhibition trends disappeared in the bar chart. All the above results illustrated that ZFSC have very good role in inhibition of human fibrosarcoma and human lung adenocarcinoma growth, which is likely related to favorable immunomodulatory effects of ZFSC.

In order to verify the immunomodulatory effect of ZFSC, ELISA was used to analyze serum IL-2, TNF-*α*, and IFN-*γ* and CD4/CD8 value, NK cell activity, and PD1 and PDL1 in spleens. The results showed that ZFSC could enhance the immune function of tumor-bearing mice. ZFSC reduced IFN-*γ* and TNF-*α* content in the serum of HT-1080 tumor-bearing mice and inhibit PD1 and PDL1. PDL1 is expressed in many tumor-associated antigen presenting cells (APCs) and tumor cells and can inhibit T cell proliferation. Negative regulation of PDL1/PD1 could reduce release of IFN-*γ* and TNF-*α* [6]. Apoptosis of tumor antigen-specific T cells is the main mechanism of PDL1/PD1 signal-mediated tumor immune escape. Blocking PDL1/PD1 signaling could be used in immunotherapy of human tumors. Therefore, the PDL1/PD1 signaling pathway is expected to become a new strategy for tumor immunotherapy. In this study, we found that the PD1 and PDL1 levels in spleens of HT-1080 tumor-bearing mice were significantly lower than those in the control group; meanwhile IFN-*γ* and TNF-*α* level in the serum of HT-1080 tumor-bearing mice were also significantly lower than those in the control group, which suggested that the antitumor mechanism of ZFSC on human fibrosarcoma could be attributed to inhibition of the PDL1/PD1 pathway, which could provide potential value to study the antitumor mechanism of ZFSC.

## Figures and Tables

**Table 1:** Inhibitory effect of ZFSC on the growth of human fibrosarcoma.

Groups	Dose (mg/kg)	Number of animals	Tumor value (mm^3^)					Tumor inhibition rate (%)
			The 6th day	The 8th day	The 10th day	The 12th day	The 14th day	
Model	—	8	58.96 ± 56.48	261.84 ± 71.78	827.13 ± 71.78	1777.54 ± 345.03	2738.46 ± 794.41	—

Positive drug	5500	8	24.89 ± 14.87	153.03 ± 82.35^*∗∗*^	564.50 ± 151.06^*∗∗*^	1650.73 ± 583.19	2736.53 ± 816.29	−3.6

ZFSC	275	8	42.36 ± 18.35	102.21 ± 49.28^*∗∗*^	420.92 ± 159.71^*∗∗*^	1034.22 ± 357.48^*∗∗*^	1632.26 ± 566.55	36.3
	550	8	23.67 ± 12.29	117.76 ± 49.53^*∗∗*^	505.40 ± 167.00^*∗∗*^	1249.96 ± 355.70^*∗*^	1899.87 ± 494.55	20.4
	1100	8	33.91 ± 17.82	104.48 ± 60.49^*∗∗*^	305.81 ± 135.94^*∗∗*^	1005.37 ± 247.63^*∗∗*^	1430.07 ± 260.28^*∗*^	40.2

Notes: ^*∗*^ *p* < 0.05 and ^*∗∗*^ *p* < 0.01, compared with the model group.

**Table 2:** Inhibitory effect of ZFSC on the growth of human lung adenocarcinoma.

Groups	Dose (mg/kg)	Number of animals	Tumor value (mm^3^)					Tumor inhibition rate (%)
			The 7th day	The 9th day	The 11th day	The 13th day	The 15th day	
Model	—	8	308.24 ± 224.52	648.62 ± 342.61	811.49 ± 418.33	1675.41 ± 770.92	2023.00 ± 917.61	—

Positive drug	5500	8	99.14 ± 59.73	274.79 ± 198.91	517.96 ± 255.54	1166.48 ± 535.88	1787.60 ± 718.99	11.8

ZFSC	275	8	242.54 ± 214.09	628.08 ± 733.08	647.32 ± 443.00	944.05 ± 473.25^*∗*^	1594.16 ± 614.72	15.0
	550	8	88.04 ± 79.69	279.67 ± 294.85	359.92 ± 325.71^*∗*^	759.22 ± 496.06^*∗∗*^	1366.88 ± 827.85	24.0
	1100	8	88.99 ± 84.81	217.70 ± 168.75^*∗*^	623.39 ± 508.89	679.60 ± 294.20^*∗∗*^	1297.82 ± 491.20	28.4

Notes: ^*∗*^ *p* < 0.05 and ^*∗∗*^ *p* < 0.01, compared with the model group.

**Table 3:** Inhibitory effect of ZFSC on the growth of human colon cancer.

Groups	Dose (mg/kg)	Number of animals	Tumor value (mm^3^)					Tumor inhibition rate (%)
			The 7th day	The 9th day	The 11th day	The 13th day	The 15th day	
Model	—	8	301.36 ± 289.54	522.50 ± 150.89	1372.86 ± 517.19	2149.74 ± 870.37	2293.42 ± 888.17	—

Positive drug	5500	8	211.20 ± 127.09	575.12 ± 277.36	959.37 ± 431.97	1880.34 ± 687.14	2086.27 ± 800.51	4.9

ZFSC	275	8	177.40 ± 92.09	487.62 ± 309.79	928.94 ± 453.68	1513.82 ± 708.16	1482.41 ± 633.38	11.8
	550	8	243.77 ± 199.55	479.30 ± 304.67	721.97 ± 271.65	1306.55 ± 448.31	1344.31 ± 491.20	17.6
	1100	8	209.68 ± 99.12	418.46 ± 146.06	773.76 ± 133.49	1292.05 ± 239.29	1392.16 ± 131.78	15.6

**Table 4:** Regulation of ZFSC on IL-2, TNF-*α*, and IFN-*γ* in serum of HT-1080 tumor-bearing mice.

Groups	Dose (mg/kg)	Number of animals	IL-2 (ng/L)	IFN-*γ* (ng/L)	TNF-*α* (ng/L)
Model	—	8	690.3 ± 26.7	808.4 ± 27.2	1167.5 ± 124.3

Positive drug	5500	8	704.6 ± 38.4	795.9 ± 64.2	1206.1 ± 99.8

ZFSC	275	8	737.1 ± 71.5	782.1 ± 53.1	1136.3 ± 49.8
	550	8	732.8 ± 28.9	639.0 ± 53.7^*∗∗*^	1071.5 ± 165.6
	1100	8	737.8 ± 47.9	634.0 ± 58.7^*∗∗*^	1023.8 ± 158.1^*∗*^

Notes: ^*∗*^ *p* < 0.05 and ^*∗∗*^ *p* < 0.01, compared with the model group.

**Table 5:** Regulation of ZFSC on IL-2, TNF-*α*, and IFN-*γ* in serum of A-549 tumor-bearing mice.

Groups	Dose (mg/kg)	Number of animals	IL-2 (ng/L)	IFN-*γ* (ng/L)	TNF-*α* (ng/L)
Model	—	8	709.6 ± 29.6	653.4 ± 59.9	993.6 ± 137.0

Positive drug	5500	8	755.3 ± 41.9^*∗*^	635.9 ± 67.5	1014.1 ± 109.6

ZFSC	275	8	769.4 ± 39.8^*∗*^	584.4 ± 24.3^*∗*^	935.2 ± 34.9
	550	8	786.5 ± 40.9^*∗∗*^	592.1 ± 43.2^*∗*^	895.9 ± 70.0
	1100	8	879.6 ± 56.6^*∗∗*^	619.0 ± 56.8	979.4 ± 99.6

Notes: ^*∗*^ *p* < 0.05 and ^*∗∗*^ *p* < 0.01, compared with the model group.

**Table 6:** Effect of ZFSC on CD4/CD8 value, NK cell activity, and PD1 and PDL1 in HT-1080 tumor-bearing mice.

Groups	Dose (mg/kg)	Number of animals	CD4/CD8	NK (nmol/L)	PD1 (pg/mL)	PDL1 (ng/L)
Model	—	8	0.087 ± 0.006	116.2 ± 12.8	82.0 ± 12.6	43.3 ± 8.8

Positive drug	5500	8	0.086 ± 0.009	117.8 ± 12.3	71.2 ± 9.0	47.2 ± 8.1

ZFSC	275	8	0.093 ± 0.011	100.4 ± 11.6^*∗*^	61.4 ± 9.8^*∗∗*^	36.0 ± 5.8
	550	8	0.092 ± 0.007	100.8 ± 24.0	69.0 ± 20.2	37.5 ± 11.1
	1100	8	0.096 ± 0.010	100.8 ± 12.9	66.1 ± 12.1^*∗*^	33.1 ± 5.0^*∗*^

Notes: ^*∗*^ *p* < 0.05 and ^*∗∗*^ *p* < 0.01, compared with the model group.

**Table 7:** Effect of ZFSC on CD4/CD8 value, NK cell activity, and PD1 and PDL1 content in A-549 tumor-bearing mice.

Groups	Dose (mg/kg)	Number of animals	CD4/CD8	NK (nmol/L)	PD1 (pg/mL)	PDL1 (ng/L)
Model	—	8	0.089 ± 0.006	79.7 ± 9.4	57.0 ± 5.4	37.6 ± 2.5

Positive drug	5500	8	0.094 ± 0.009	86.7 ± 13.7	59.9 ± 4.6	37.1 ± 2.6

ZFSC	275	8	0.089 ± 0.005	82.1 ± 8.8	60.6 ± 8.7	37.3 ± 3.0
	550	8	0.087 ± 0.010	86.2 ± 11.6	65.8 ± 10.4	36.6 ± 4.2
	1100	8	0.087 ± 0.010	84.6 ± 6.7	65.0 ± 5.9	38.4 ± 3.3
